# Nonalcoholic fatty liver disease accelerates kidney function decline in patients with chronic kidney disease: a cohort study

**DOI:** 10.1038/s41598-018-23014-0

**Published:** 2018-03-16

**Authors:** Hye Ryoun Jang, Danbee Kang, Dong Hyun Sinn, Seonhye Gu, Soo Jin Cho, Jung Eun Lee, Wooseong Huh, Seung Woon Paik, Seungho Ryu, Yoosoo Chang, Tariq Shafi, Mariana Lazo, Eliseo Guallar, Juhee Cho, Geum-Youn Gwak

**Affiliations:** 1 Department of Medicine, Samsung Medical Center, Sungkyunkwan University School of Medicine, Kangbuk Samsung Hospital, Seoul, South Korea; 2 Department of Clinical Research Design and Evaluation, SAIHST, Sungkyunkwan University, Kangbuk Samsung Hospital, Seoul, South Korea; 3 Center for Clinical Epidemiology, Samsung Medical Center, Sungkyunkwan University, Kangbuk Samsung Hospital, Seoul, South Korea; 4 Center for Health Promotion, Samsung Medical Center, Kangbuk Samsung Hospital, Seoul, South Korea; 5 Department of Clinical Pharmacology and Therapeutics, Samsung Medical Center, Samsung Biomedical Research Institute, Sungkyunkwan University School of Medicine, Kangbuk Samsung Hospital, Seoul, South Korea; 6 Center for Total Health Studies, Kangbuk Samsung Hospital, Seoul, South Korea; 7 Division of Nephrology, Department of Medicine, Johns Hopkins University School of Medicine, Johns Hopkins Medical Institutions, Baltimore, Maryland USA; 8 Departments of Epidemiology and Medicine and Welch Center for Prevention, Epidemiology and Clinical Research, Johns Hopkins Medical Institutions, Baltimore, Maryland USA

## Abstract

This study aimed to investigate the association of nonalcoholic fatty liver disease (NAFLD) and its severity with the decline in kidney function in patients with chronic kidney disease (CKD). We conducted a cohort study of 1,525 CKD patients who underwent repeated health check-up examinations from January 2003 through December 2013. NAFLD was diagnosed by ultrasonography and its severity was assessed by the NAFLD fibrosis score. At baseline, the prevalence of NAFLD was 40.9%, and the mean estimated glomerular filtration rate (eGFR) was 59.1 ml/min/1.73 m^2^. The average follow-up was 6.5 years. The age- and sex-adjusted decline in eGFR was greater in patients with NAFLD (−0.79% per year, 95% CI −1.31%, −0.27%) compared to those without it (0.30%, 95% CI −0.14%, 0.76%; p = 0.002). In multivariable adjusted models, the average difference in annual percent change in decline in eGFR comparing patients with NAFLD to those without NAFLD was −1.06% (−1.73%, −0.38%; p = 0.002). The decline in eGFR associated with NAFLD was greater in patients with higher NAFLD fibrosis score, in those with proteinuria or with low eGFR at baseline ( <45 ml/min/1.73 m^2^), and in those who were smokers and hypertensive. Therefore, NAFLD is independently associated with CKD progression.

## Introduction

Chronic kidney disease (CKD) is associated with high morbidity and mortality, and is rapidly becoming a major burden to the global health care system^1,2^. The global prevalence of CKD was estimated at 10.4–13.4%^3^. End-stage renal disease (ESRD), the last stage of CKD requiring renal replacement therapy, is rapidly increasing worldwide and expected to further increase in the next decade^4^. There is a substantial interest in identifying novel and potentially reversible causes of progression from early stages of CKD to ESRD.

Nonalcoholic fatty liver disease (NAFLD), characterized by excessive hepatic fat accumulation without significant alcohol intake, use of medications causing fatty liver, or other traditional causes of fatty liver, is the most common cause of chronic liver disease worldwide, especially in developed countries^5,6^. The global prevalence of NAFLD was estimated at 25.2%^7^. Furthermore, the consequences of NAFLD extend beyond the liver^8^. NAFLD is associated with increased risk of type 2 diabetes and cardiovascular disease^9–11^, and with increased prevalence and incidence of CKD^12–14^. No study, however, has evaluated the role of NAFLD on the decline in kidney function among patients who already have reduced kidney function (CKD stage 3 or more). NAFLD is frequently accompanied by multiple traditional risk factors for CKD progression including obesity and diabetes, but evidence supporting the independent role of NAFLD on CKD progression is still lacking.

We thus investigated the association of NAFLD and its severity with the longitudinal decline in kidney function in a cohort of CKD patients, adjusting for traditional risk factors for CKD.

## Results

The mean (standard deviation, SD) age of study patients was 60.8 (11.3) years, and the prevalence of NAFLD at baseline was 40.9% (Table 1). Compared to patients without NAFLD, those with NAFLD were more likely to be young, male, smokers, and had a higher level of body mass index (BMI), total cholesterol, and other metabolic risk factors. The mean estimated glomerular filtration rate (eGFR) at baseline was 59.1 ml/min/1.73 m^2^ (26.2% patients had proteinuria ≥2+ on urinalysis). While eGFR at baseline was higher in patients with NAFLD compared to those without NAFLD at baseline (61.8 vs. 57.3 ml/min/1.73 m^2^, p < 0.001), patients with NAFLD had higher proportion of proteinuria than those without NAFLD (30.2 vs. 23.5%, p = 0.004).

**Table 1:** Baseline characteristics of study population.

Characteristics	Overall	Non-Alcoholic Fatty Liver Disease (NAFLD) Status		p value
		No	Yes	
Number of patients	1,525	902	623	
Age, years	60.8 (11.3)	61.4 (11.5)	59.9 (10.9)	0.009
Sex				<0.001
Male	1,065 (69.8)	567 (62.9)	498 (79.9)	
Female	460 (30.2)	335 (37.1)	125 (20.1)	
BMI, kg/m^2^	24.8 (3.0)	23.7 (2.7)	26.4 (2.7)	<0.001
Smoking				<0.001
Never	742 (48.7)	485 (53.8)	257 (41.3)	
Past	216 (14.2)	111 (12.3)	105 (16.9)	
Current	255 (16.7)	122 (13.5)	133 (21.4)	
Missing	312 (20.5)	184 (20.4)	128 (20.6)	
Moderate alcohol consumption	868 (56.9)	475 (52.7)	393 (63.1)	<0.001
Fasting glucose, mg/dl	104.1 (30.1)	99.6 (24.8)	112.1 (34.9)	<0.001
Hemoglobin A1c, %	5.9 (1.1)	5.7 (1.0)	6.2 (1.2)	<0.001
Use of antidiabetic medications	255 (16.7)	127 (14.1)	128 (20.6)	0.001
Diabetes	359 (23.5)	155 (17.2)	204 (32.7)	<0.001
Systolic blood pressure, mmHg	126.5 (18.8)	125.4 (19.1)	128.1 (18.3)	0.006
Use of antihypertensive medications	731 (48.0)	391 (43.4)	341 (54.7)	<0.001
Hypertension	913 (59.9)	502 (55.7)	411 (66.0)	<0.001
Triglycerides, mg/dl	125 (94–179)	110 (83–149)	151 (114–213)	<0.001
Total cholesterol, mg/dl	201.8 (39.2)	199.1 (38.9)	205.8 (39.2)	0.001
LDL cholesterol, mg/dl	131.1 (35.3)	128.8 (35.0)	134.4 (35.6)	0.002
HDL cholesterol, mg/dl	50.1 (13.8)	52.8 (15.0)	46.2 (10.6)	<0.001
Lipid lowering medications	85 (5.6)	46 (5.1)	39 (6.3)	0.33
Hyperlipidemia	630 (41.3)	285 (31.6)	345 (55.4)	<0.001
ALT, U/L	26.2 (16.9)	21.2 (11.8)	33.5 (20.2)	<0.001
AST, U/L	26.0 (13.8)	23.7 (8.5)	29.2 (18.6)	<0.001
GGT, U/L	37.3 (39.3)	29.9 (30.6)	48.1 (47.2)	<0.001
Estimated GFR, ml/min/1.73 m^2^	59.1 (17.3)	57.3 (16.8)	61.8 (17.6)	<0.001
Protein ≥2+ on urinalysis	400 (26.2)	212 (23.5)	188 (30.2)	0.004

Values are mean (SD), number (%), or median (interquartile range).

ALT, alanine aminotransferase; AST, aspartate aminotransferase; BMI, body mass index; GFR, glomerular filtration rate; GGT, gamma-glutamyltransferase; LDL, low-density lipoprotein; HDL, high-density lipoprotein.

The average duration of follow-up was 6.5 years (maximum 13.6 years; average number of visits per participant 6.3). During follow-up, the age- and sex-adjusted decline in eGFR was greater in patients with NAFLD (−0.79% per year, 95% CI −1.31%, −0.27%) compared to those without it (0.30%, 95% CI −0.14%, 0.76%; p = 0.002).

After adjustment for age, sex, and year of visit, the average difference in annual percent change of eGFR between patients with NAFLD and those without NAFLD was −1.09% (−1.89%, −0.27%; p = 0.009; Table 2). The results did not materially change after adjusting for confounders or potential metabolic mediators. The multivariable adjusted average difference in annual percent changes in eGFR comparing patients with low NAFLD fibrosis score (NFS) ( < −1.455) or intermediate to high NFS ( ≥ −1.455) and those without NAFLD were 0.01% (−0.74%, 0.99%) and −2.12% (−2.93%, −1.31%), respectively (Table 2).

**Table 2:** Average difference in annual percent change in estimated glomerular filtration rate (eGFR) according to the presence and severity of nonalcoholic fatty liver disease (NAFLD) at baseline (n = 1,525).

	Average difference in annual percent change in eGFR (%)		
	Model 1 HR (95% CI)	Model 2 HR (95% CI)	Model 3 HR (95% CI)
**NAFLD**			
No NAFLD	0.00 (reference)	0.00 (reference)	0.00 (reference)
NAFLD	−1.09 (−1.77, −0.41)	−1.08 (−1.76, −0.40)	−1.06 (−1.73, −0.38)
p value	0.002	0.002	0.002
**NAFLD severity**			
No NAFLD	0.00 (reference)	0.00 (reference)	0.00 (reference)
NAFLD with NFS <−1.455	0.04 (−0.83, 0.91)	0.05 (−0.82, 0.92)	0.01 (−0.74, 0.99)
NAFLD with NFS ≥ −1.455	−2.12 (−2.94, −1.30)	−2.13 (−2.94, −1.30)	−2.12 (−2.93, −1.31)
p value	<0.001	<0.001	<0.001

eGFR, estimated glomerular filtration rate; CI, confidence interval; NAFLD, non-alcoholic fatty liver disease; NFS, NAFLD fibrosis score.

Model 1: Adjusted for age, sex, and year of visit

Model 2: Further adjusted for baseline smoking status (never, former, current, and missing), alcohol intake (none and moderate), and body mass index.

Model 3: Further adjusted for hypertension, diabetes, hyperlipidemia, systolic blood pressure, hemoglobin A1c, LDL cholesterol, and triglycerides (log_e_-transformed).

For the NFS analyses, the models were not adjusted for age, body mass index, and diabetes as these factors are included in the calculation of the NFS.

**Figure 1:**
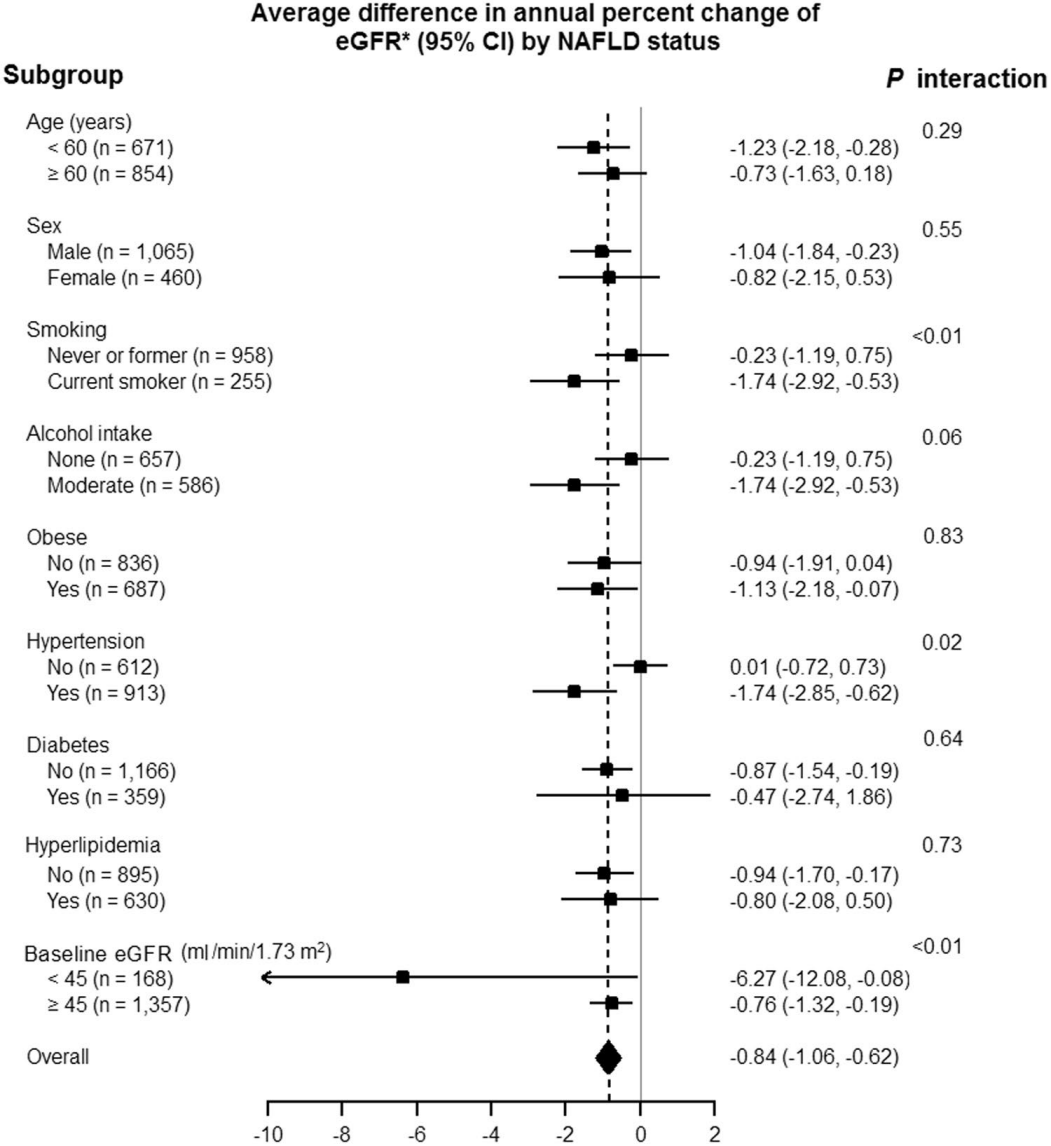
Average difference in annual percent change in estimated glomerular filtration rate (eGFR) in predefined subgroups of CKD patients by baseline nonalcoholic fatty liver disease (NAFLD) status (n = 1,326). *Expected annual percent changes were derived from linear mixed effects models for log-transformed eGFR adjusted for baseline age, sex, year of visit, baseline smoking status (never, former, current, and missing), alcohol (none and moderate), body mass index, hypertension, diabetes, hyperlipidemia, systolic blood pressure, hemoglobin A1c, LDL cholesterol, and triglycerides (log_e_-transformed).

The association between NAFLD and CKD progression was also observed in patients with only eGFR < 60 ml/min/1.73 m^2^, with only proteinuria, or with both at baseline (Table 3). The association between NAFLD and the decline in eGFR was consistent across most subgroups (Fig. 1), although the decline in eGFR associated with NAFLD appeared to be stronger among patients who were current smokers (p for interaction < 0.01), hypertensive (p for interaction = 0.02), and those with lower eGFR at baseline (p for interaction < 0.01).

**Table 3:** Average difference in annual percent change in estimated glomerular filtration rate (eGFR) according to the presence and severity of nonalcoholic fatty liver disease (NAFLD) and CKD criteria (n = 1,525).

Baseline status	Average difference in annual percent changes in eGFR (%)		
	Model1	Model2	Model3
**eGFR < 60 ml/min/1**.**73 m**^**2**^			
**NAFLD**			
No NAFLD	0.00 (reference)	0.00 (reference)	0.00 (reference)
NAFLD	−0.47 (−1.10, 0.16)	−0.47 (−1.09, 0.17)	−0.41 (−1.02, 0.21)
p value	0.14	0.16	0.20
**NAFLD severity**			
No NAFLD	0.00 (reference)	0.00 (reference)	0.00 (reference)
NAFLD with NFS < −1.455	0.41 (−0.41, 1.23)	0.42 (−0.40, 1.24)	0.54 (−0.26, 1.35)
NAFLD with NFS ≥ −1.455	−1.27 (−2.04, −0.50)	−1.27 (−2.03, −0.50)	−1.27 (−2.02, −0.52)
p for trend	0.007	0.007	0.008
**Proteinuria**			
**NAFLD**			
No NAFLD	0.00 (reference)	0.00 (reference)	0.00 (reference)
NAFLD	−1.17 (−2.35, 0.02)	−1.19 (−2.37, 0.01)	−1.21 (−2.39, −0.01)
p value	0.055	0.052	0.047
**NAFLD severity**			
No NAFLD	0.00 (reference)	0.00 (reference)	0.00 (reference)
NAFLD with NFS < −1.455	−0.39 (−1.77, 1.02)	−0.40 (−1.79, 1.01)	−0.48 (−1.88, 0.94)
NAFLD with NFS ≥ −1.455	−2.28 (−3.69, −0.84)	−2.28 (−3.70, −0.83)	−2.24 (−3.67, −0.79)
p for trend	0.004	0.004	0.004
**Both eGFR < 60 and proteinuria**			
**NAFLD**			
No NAFLD	0.00 (reference)	0.00 (reference)	0.00 (reference)
NAFLD	−7.16 (−15.50, 2.01)	−7.16 (−15.71, 2.01)	−7.08 (−15.32, 1.97)
p value	0.12	0.12	0.12
**NAFLD severity**			
No NAFLD	0.00 (reference)	0.00 (reference)	0.00 (reference)
NAFLD with NFS < −1.455	−4.22 (−17.34, 11.00)	−4.40 (−17.63, 10.96)	−4.66 (−17.93, 10.76)
NAFLD with NFS ≥ −1.455	−8.01 (−16.82, 1.74)	−8.06 (−16.96, 1.79)	−8.02 (−16.98, 1.90)
p for trend	0.10	0.10	0.11
**p for interaction**	0.003	0.003	0.003

eGFR, estimated glomerular filtration rate; NAFLD, non-alcoholic fatty liver disease; NFS, NAFLD fibrosis score.

Model 1: Adjusted for age, sex and year of visit

Model 2: Further adjusted for baseline smoking status (never, former, current, and missing), alcohol intake (none and moderate), and body mass index.

Model 3: Further adjusted for hypertension, diabetes, hyperlipidemia, systolic blood pressure, hemoglobin A1c, LDL cholesterol, and triglycerides (log_e_-transformed).

For the NFS analyses, the models were not adjusted for age, body mass index, and diabetes as these factors are included in the calculation of the NFS.

Among patients with eGFR < 45 ml/min/1.73 m^2^ at baseline (Supplementary Table S1), the multivariable adjusted average difference in annual percent change of eGFR between patients with NAFLD and those without NAFLD was −5.61% (−11.43%, 0.59%; p = 0.075; Table 4).

**Table 4:** Average difference in annual percent change in estimated glomerular filtration rate (eGFR) in CKD patients with eGFR lower than 45 ml/min/1.73 m^2^ According to baseline nonalcoholic fatty liver disease (NAFLD) status (n = 168).

Annual percent change in eGFR	NAFLD		p-value
	No (n = 116)	Yes (n = 52)	
Average difference			
Model 1	0.00 (reference)	−6.31 (−12.08, −0.16)	0.044
Model 2	0.00 (reference)	−6.33 (−12.13, −0.14)	0.045
Model 3	0.00 (reference)	−5.61 (−11.43, 0.59)	0.075

eGFR, estimated glomerular filtration rate; NAFLD, non-alcoholic fatty liver disease.

Model 1: Adjusted for age, sex, and year of visit.

Model 2: Further adjusted for baseline smoking status (never, former, current, and missing), alcohol intake (none and moderate), and body mass index.

Model 3: Further adjusted for hypertension, diabetes, hyperlipidemia, systolic blood pressure, hemoglobin A1c, LDL cholesterol, and triglycerides (log_e_-transformed).

## Discussion

In this large longitudinal study, NAFLD was an independent risk factor associated with the progression of CKD. The association was stronger in patients with more advanced NAFLD, as indicated by a higher fibrosis score, in those with lower eGFR, in those with proteinuria, and in patients who were current smokers, and hypertensive. Furthermore, the association did not appear to be mediated by traditional risk factors associated with NAFLD. To our knowledge, this is the first report to implicate NAFLD as an important risk factor for CKD progression. Further research needs to consider the clinical impact of screening for and managing NAFLD in CKD patients.

The control of CKD progression is still an unresolved issue^4,15^. Few specific treatments delay the progression of CKD, especially in patients with advanced CKD, so that identifying risk factors or mediators other than diabetes or hypertension is critically important. NAFLD is a common comorbidity in CKD patients. Although several studies have evaluated the association of NAFLD and CKD, most of them were cross-sectional and did not address the role of NAFLD in CKD progression^16–18^. Similarly, several studies have identified an association between NAFLD and proteinuria^16–21^, but a longitudinal association of NAFLD with kidney function decline in CKD patients had not been evaluated.

Several mechanisms may explain a role of NAFLD in CKD progression. NAFLD is associated with the activation of the nuclear factor-κB and the C-Jun-N-terminal kinase pathways, which enhance transcription of several proinflammatory genes that amplify systemic chronic inflammation and insulin resistance, respectively^22–26^. These pathways may facilitate CKD progression by augmenting intrarenal immunologic inflammatory responses in CKD, especially in glomerulonephritis where the main pathogenic mechanism is immunologic reaction^27^. The final common pathologic features in CKD progression are glomerulosclerosis and tubular atrophy accompanied by interstitial fibrosis^28–30^. Pro-inflammatory milieu in NAFLD may accelerate the development of these pathological features in patients with CKD even in patients in whom the initial pathogenic mechanism was not immunologic.

In our study, the association of NAFLD with kidney function decline was stronger in current smokers, and in those who were hypertensive or who had baseline eGFR < 45 ml/min/1.73 m^2^. This finding suggests specific subgroups may benefit most from screening, prevention and management of NAFLD. The importance of smoking cessation and hypertension control for attenuating CKD progression has been documented in many studies and emphasized in the Kidney Disease Outcomes Quality Initiative guidelines^31–34^. The interplay between NAFLD and traditional risk factors of CKD, however, has not been fully elucidated^35–38^. Further studies should focus on the interaction between NAFLD and traditional risk factors for CKD progression.

Several limitations need to be considered in the interpretation of our findings. First, we did not have information on the primary cause of CKD. However, we had detailed clinical and laboratory information on comorbidities including diabetes and hypertension, medications, anthropometry, and blood chemistry that allowed for adjusting for multiple confounders and evaluating potential mediators. Second, proteinuria was not measured using a quantitative assay. Thus, we excluded trace or 1 + protein on urinalysis from the proteinuria subgroup because of the possibility of false positives. Third, NAFLD was measured by ultrasound after exclusion of secondary causes for fatty liver. Although the accuracy of ultrasound for establishing the presence of fatty liver is high, it is subject to measurement error^6,39^. Similarly, we used eGFR as an outcome, which has inherent limitations derived from using creatinine as a filtration marker. Finally, this study was based on Korean patients in health screening exams, and may not be generalizable to other settings or to other ethnicities.

Screening for NAFLD in CKD patients is not routinely performed and there is no specific guideline for screening NAFLD in CKD patients^34^. Our data suggest that routine screening for NAFLD needs to be contemplated among CKD patients, although additional studies are required to determine the approach and the clinical management of NAFLD in CKD patients. Most treatment strategies for NAFLD involve lifestyle modification such as weight reduction, hypocaloric diet, increase physical activity, smoking cessation, and treatment of associated metabolic risk factors (diabetes, hypertension, and dyslipidemia)^5^. Future studies will also need to evaluate whether NAFLD resolution can reduce the risk of CKD progression independently of traditional risk factors^40^.

In summary, NAFLD was associated with declining kidney function in CKD patients independently of established risk factors, and the association was stronger in patients with advanced NAFLD, and in those with proteinuria or with eGFR lower than 45 ml/min/1.73 m^2^. Our findings suggest that NAFLD may contribute to CKD progression and may help identify patients with a higher risk of rapid progression. CKD patients may benefit from screening and management of NAFLD, which warrants prospective validation.

## Methods

### Study population

**Figure 2:**
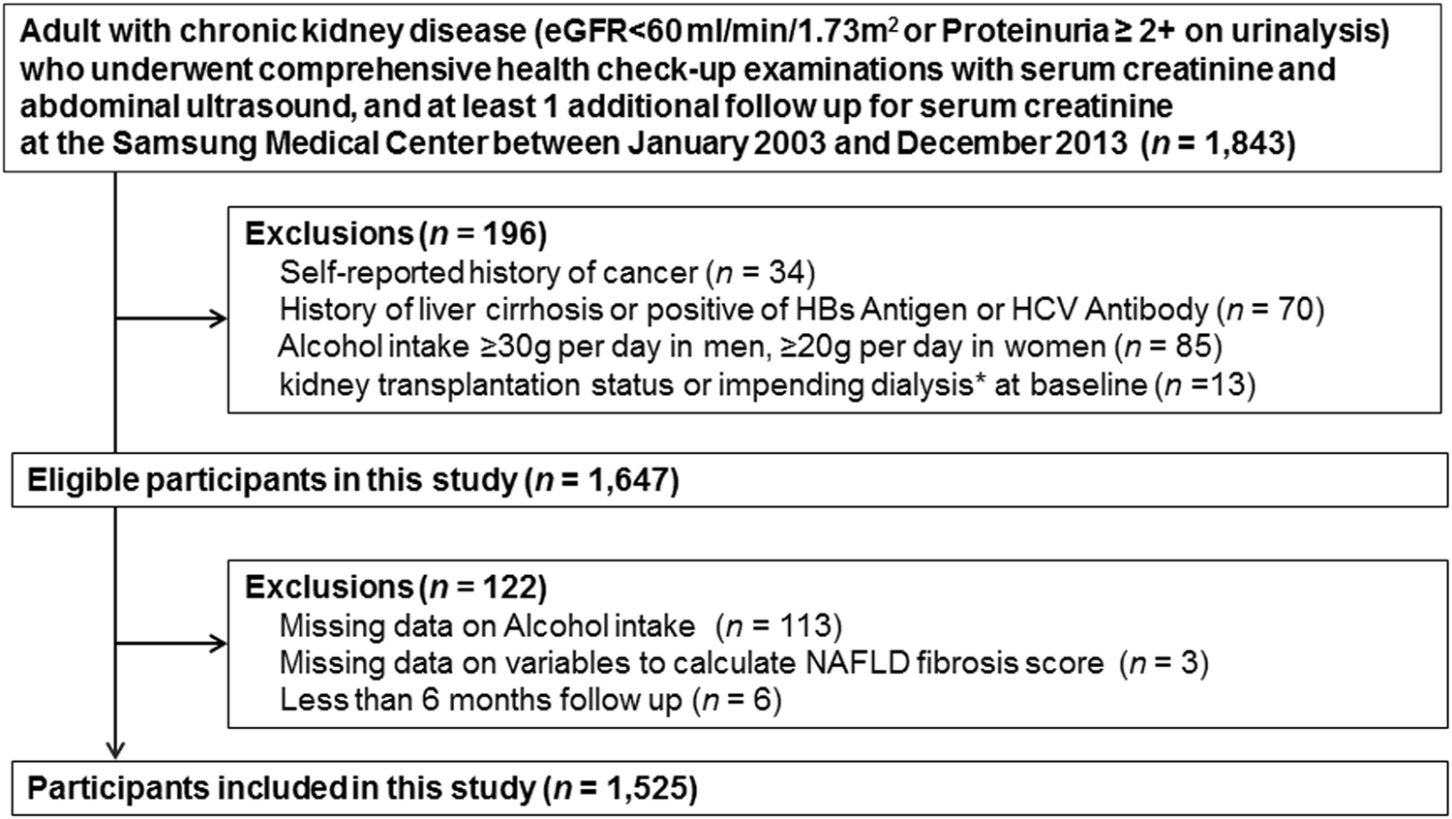
Flow chart of study patients (n = 1,326). *Impending dialysis represents patients who started dialysis within 1 year after the baseline health check-up examination.

We conducted a retrospective cohort analysis of men and women 18 years of age or older who underwent a comprehensive health screening examination at the Samsung Medical Center Health Promotion Center in Seoul, South Korea, from January 2003 to December 2013 (Fig. 2). Since our objective was to evaluate the longitudinal association between NAFLD and change of eGFR among patients with CKD, the analysis was restricted to subjects who had CKD (eGFR < 60 ml/min/1.73 m^2^ or proteinuria ≥2 + on urinalysis), and underwent an abdominal ultrasound (US) at baseline, and had at least 1 additional follow up for serum creatinine (n = 1,843). We then excluded patients who had any of the following conditions at baseline: history of cancer (n = 34), history of liver cirrhosis, positive hepatitis B surface antigen, or hepatitis C virus antibodies (n = 70), alcohol intake ≥ 30 g/day in men or ≥20 g/day in women (n = 85), or had received a kidney transplant or started dialysis within 1 year after the baseline examination (n = 13). We also excluded patients who had missing information on alcohol intake (n = 113), NFS (n = 3), or less than 6 months follow up (n = 6). Since study patients could have more than one exclusion criteria, the final sample size was 1,525 (1,065 men and 460 women). The Institutional Review Board of the Samsung Medical Center approved this study and waived the requirement for informed consent as we used only de-identified data routinely collected during health screening visits. All methods were performed in accordance with the relevant guidelines and regulations.

### Data collection

At each visit, demographic characteristics, smoking status, alcohol consumption, medical history, and medication use were collected through standardized, self-administered questionnaires. Smoking status was categorized into never, former, or current smoker. Alcohol consumption was categorized into none or moderate (<30 g/day in men and <20 g/day in women). Height, weight, and sitting blood pressure were measured by trained nurses. BMI was calculated as weight in kilograms divided by height in meters squared.

Serum creatinine was measured with the kinetic alkaline picrate method (Jaffe method) using automated chemistry analyzers Hitachi 7600 (Hitachi, Tokyo, Japan) from 2003 to 2009 and Modular DP (Roche, Basel, Switzerland) from 2009 to 2013. Serum glucose was measured by the hexokinase/glucose-6-phosphate dehydrogenase method. Hemoglobin A1c (HbA1c) was measured by high performance liquid chromatography. Aspartate aminotransferase (AST), alanine aminotransferase (ALT), and gamma-glutamyltransferase (GGT) were measured following the International Federation of Clinical Chemistry method. Urine protein was measured semi-quantitatively by urine dipstick using urine chemistry analyzers, Cliniteck Atlas (Siemens, Munich, Germany) or Urisys 2400 (Roche). The Department of Laboratory Medicine and Genetics at Samsung Medical Center has participated in several proficiency testing programs operated by the Korean Association of Quality Assurance for Clinical Laboratory, the Asian Network of Clinical Laboratory Standardization and Harmonization, and the College of American Pathologists.

### Abdominal US

Abdominal US imaging was performed using LogiQ E9 (GE Healthcare, Milwaukee, Wisconsin, USA), iU22 xMatrix (Philips Medical Systems, Cleveland, Ohio, USA) or ACUSON Sequoia 512 (Siemens, Issaquah, Washington, USA) equipments by experienced radiologists unaware of the study aims. Images were captured in a standard fashion with the patient in the supine position with the right arm raised above the head. An US diagnosis of fatty liver was made based on standard criteria, including parenchymal brightness, liver-to-kidney contrast, deep beam attenuation and bright vessel walls^41,42^. Since we had already excluded patients with excessive alcohol use (≥30 g/day for men and ≥20 g/day for women) as well as other identifiable causes of fatty liver at baseline as described in the exclusion criteria, fatty liver was considered NAFLD.

Among individuals with NAFLD, we used the NFS to assess severity of fibrosis. The NFS was calculated as −1.675 + 0.037 × age (years) + 0.094 × BMI (kg/m^2^) + 1.13 × impaired fasting glucose/diabetes (yes = 1, no = 0) + 0.99 × AST/ALT ratio − 0.013 × platelet count (×10^9^/l) − 0.66 × albumin (g/dl)^43^. Based on NFS, patients with NAFLD were classified as high-intermediate (NFS ≥ −1.455) and low probability (NFS < −1.455) of advanced fibrosis.

### Estimated glomerular filtration rate and proteinuria

EGFR was calculated from serum creatinine using the CKD epidemiology collaboration (CKD-EPI) equation^44^: for women with serum creatinine ≤ 0.7, eGFR was calculated as 144 × (serum creatinine/0.7)^−0.329^× (0.993)^age^; for women with serum creatinine >0.7, eGFR was calculated as 144 × (serum creatinine/0.7)^−1.209^ × (0.993)^age^; for men with serum creatinine ≤ 0.9, eGFR was calculated as 141 × (serum creatinine/0.9)^−0.411^ × (0.993)^age^; and for men with serum creatinine > 0.9, eGFR was calculated as 141 × (serum creatinine/0.9)^−1.209^ × (0.993)^age^. Significant proteinuria was defined as protein ≥2+ on urinalysis. CKD was defined as eGFR < 60 ml/min/1.73 m^2^ or protein ≥2+ on urinalysis at the baseline examination. The degree of CKD progression was evaluated as the average annual percent change in eGFR from baseline eGFR. For the severity of CKD, patients were further divided into two groups using a cut-off value of eGFR ≥45 ml/min/1.73 m^2^ vs. <45 ml/min/1.73 m^2^ (dividing G3a and G3b) at baseline.

### Statistical analysis

We compared serial changes in eGFR among CKD patients with or without NAFLD at baseline using linear mixed models for longitudinal data with random intercepts and random slopes^45^. To account for correlations in eGFR from repeated measurements over time in the same participant, the main analyses consisted of two-level linear mixed models for longitudinal data. We modeled linear trajectories in eGFR with time at the first level, and variations in eGFR trajectories across participants at the second level. We used log_e_-transformed (eGFR) as the outcome and we estimated the average difference in annual percent change in eGFR (with 95% confidence intervals [CI]) comparing patients with NAFLD to those without NAFLD at baseline. The 95% CIs were obtained from linear mixed models with different intersecting linear trends during follow-up time, interactions between linear time and baseline NAFLD status, and random variations in linear time among participants.

We used three models with increasing degrees of adjustment to account for potential confounding factors at baseline. Model 1 adjusted for age, sex, and year of visit. Model 2 further adjusted for smoking (never, former, current, and missing), alcohol consumption (none and moderate), and BMI. In addition, to evaluate potential mediation of the association between NAFLD and eGFR changes by metabolic factors, model 3 further adjusted for hypertension, diabetes, hyperlipidemia, systolic blood pressure, HbA1c, low-density lipoprotein (LDL) cholesterol, and triglycerides.

We conducted sensitivity analyses depending on CKD criteria at baseline: eGFR < 60 ml/min/1.73 m^2^ only, proteinuria only, and both. In addition, we performed stratified analyses to evaluate if the association of NAFLD with CKD progression differed in pre-specified subgroups defined by age (<60 vs. ≥ 60 years), sex, smoking (never or former vs. current), alcohol drinking (none vs. moderate), obese (defined as BMI ≥ 25 kg/m^2^), hypertension (defined as systolic blood pressure ≥ 140 mmHg, diastolic blood pressure ≥ 90 mmHg, or the use of antihypertensive medication), diabetes (defined as fasting serum glucose ≥ 126 mg/dl, hemoglobin A1c ≥ 6.5%, or the use of antidiabetic medication), hyperlipidemia (defined as high-density lipoprotein (HDL)-cholesterol < 40 mg/dl in men or < 50 mg/dl in women, triglycerides ≥ 150 mg/dl, or the use of lipid-lowering medication), or baseline eGFR (<45 vs. ≥ 45 ml/min/1.73 m^2^). All reported *P* values were two-sided and the significance level was set at 0.05. Statistical analyses were performed using Stata version 14 (Stata Corp., College Station, Texas).

## Electronic supplementary material

Supplementary Table
