# Author Correction: Nonalcoholic fatty liver disease accelerates kidney function decline in patients with chronic kidney disease: a cohort study

**DOI:** 10.1038/s41598-021-90150-5

**Published:** 2021-05-21

**Authors:** Hye Ryoun Jang, Danbee Kang, Dong Hyun Sinn, Seonhye Gu, Soo Jin Cho, Jung Eun Lee, Wooseong Huh, Seung Woon Paik, Seungho Ryu, Yoosoo Chang, Tariq Shafi, Mariana Lazo, Eliseo Guallar, Juhee Cho, Geum-Youn Gwak

**Affiliations:** 1 Department of Medicine, Samsung Medical Center, Sungkyunkwan University School of Medicine, Seoul, South Korea; 2 Department of Clinical Research Design and Evaluation, SAIHST, Sungkyunkwan University, Seoul, South Korea; 3 Center for Clinical Epidemiology, Samsung Medical Center, Sungkyunkwan University, Seoul, South Korea; 4 Center for Health Promotion, Samsung Medical Center, Seoul, South Korea; 5 Department of Clinical Pharmacology and Therapeutics, Samsung Medical Center, Samsung Biomedical Research Institute, Sungkyunkwan University School of Medicine, Seoul, South Korea; 6 Center for Total Health Studies, Kangbuk Samsung Hospital, Seoul, South Korea; 7 Division of Nephrology, Department of Medicine, Johns Hopkins University School of Medicine, Johns Hopkins Medical Institutions, Baltimore, Maryland USA; 8 Departments of Epidemiology and Medicine and Welch Center for Prevention, Epidemiology and Clinical Research, Johns Hopkins Medical Institutions, Baltimore, Maryland USA

Correction to: *Scientific Reports* 10.1038/s41598-018-23014-0, published online 16 March 2018

The original version of this Article contained a repeated error in Affiliations 1,2,3,4 and 5 where “Kangbuk Samsung Hospital” was incorrectly given as the Organisational name.

The correct affiliations are listed below:

Affiliation 1

Department of Medicine, Samsung Medical Center, Sungkyunkwan University School of Medicine, Seoul, South Korea

Affiliation 2

Department of Clinical Research Design and Evaluation, SAIHST, Sungkyunkwan University, Seoul, South Korea

Affiliation 3

Center for Clinical Epidemiology, Samsung Medical Center, Sungkyunkwan University, Seoul, South Korea

Affiliation 4

Center for Health Promotion, Samsung Medical Center, Seoul, South Korea

Affiliation 5

Department of Clinical Pharmacology and Therapeutics, Samsung Medical Center, Samsung Biomedical Research Institute, Sungkyunkwan University School of Medicine, Seoul, South Korea

The original Article has been corrected.

