# Supplementary material for: Nonalcoholic fatty liver disease accelerates kidney function decline in patients with chronic kidney disease: a cohort study
Source: Sci Rep. 2018 Mar 16;8:4718. doi: 10.1038/s41598-018-23014-0 (PMC5856790; doi:10.1038/s41598-018-23014-0)
Supplement: Supplementary file 1 — Supplementary Table [file 41598_2018_23014_MOESM1_ESM.docx]

**Nonalcoholic fatty liver disease accelerates kidney function decline in patients with chronic kidney disease: a cohort study**

Hye Ryoun Jang^1^, Danbee Kang^2^, Dong Hyun Sinn^1^, Seonhye Gu^3^, Soo Jin Cho^4^, Jung Eun Lee^1^, Wooseong Huh^1,5^, Seung Woon Paik^1^, Seungho Ryu^6^, Yoosoo Chang^6^, Tariq Shafi^7,8^, Mariana Lazo^8^, Eliseo Guallar^3,8^, Juhee Cho^2,3,8^, Geum-Youn Gwak^1^

**Supplementary Table S1.** Baseline characteristics of CKD patients with eGFR < 45 ml/min/1.73 m^2^

| **Characteristics** | **Overall** | **Non-Alcoholic Fatty Liver Disease (NAFLD) status** | | **p value** |
| --- | --- | --- | --- | --- |
|  |  | **No** | **Yes** |  |
| Number of participants | 168 | 116 | 52 |  |
| Age, years | 64.8 (11.3) | 65.4 (10.9) | 63.1 (12.0) | 0.21 |
| Sex |  |  |  | 0.052 |
| Male | 108 (64.3) | 69 (59.5) | 39 (75.0) |  |
| Female | 60 (35.7) | 47 (40.5 | 13 (25.0) |  |
| BMI, kg/m^2^ | 24.9 (3.1) | 24.1 (3.0) | 26.5 (2.6) | <0.001 |
| Smoking |  |  |  | 0.29 |
| Never | 87 (51.8) | 65 (56.0) | 22 (42.3) |  |
| Past | 16 (9.5) | 10 (8.6) | 6 (11.5) |  |
| Current | 24 (14.3) | 17 (14.7) | 7 (13.5) |  |
| Missing | 41 (24.4) | 24 (20.7) | 17 (32.7) |  |
| Moderate alcohol consumption | 84 (50.0) | 54 (46.6) | 30 (57.7) | 0.18 |
| Fasting glucose, mg/dl | 103.7 (23.9) | 103.4 (26.4) | 104.2 (17.3) | 0.84 |
| Hemoglobin A1c | 6.0 (1.1) | 5.97 (1.1) | 6.1 (0.9) | 0.45 |
| Use of antidiabetic medications | 120 (28.6) | 31 (26.7) | 9 (17.3) | 0.19 |
| Diabetes | 50 (29.8) | 36 (31.0) | 14 (26.9) | 0.59 |
| Systolic blood pressure, mm Hg | 131.8 (20.7) | 131.0 (21.4) | 133.5 (19.3) | 0.46 |
| Use of antihypertensive medications | 120 (72.4) | 85 (73.3) | 35 (67.3) | 0.43 |
| Hypertension | 136 (81.0) | 97 (83.6) | 39 (75.0) | 0.19 |
| Triglyceride, mg/dl | 131 (96.5-199) | 123.5 (88.5-162.4) | 183.5 (112-234.5) | <0.001 |
| Total cholesterol, mg/dl | 190.9 (42.8) | 186.3 (42.3) | 201.2 (42.4) | 0.036 |
| LDL cholesterol, mg/dl | 120.8 (37.5) | 117.1 (36.7) | 128.8 (38.2) | 0.06 |
| HDL cholesterol, mg/dl | 46.2 (14.2) | 47.4 (15.2) | 43.7 (11.6) | 0.12 |
| Use of lipid-lowering medications | 14 (8.3) | 12 (10.3) | 2 (3.9) | 0.14 |
| Hyperlipidemia | 87 (51.8) | 53 (45.7) | 34 (65.4) | 0.018 |
| Estimated GFR, ml/min/1.73 m^2^ | 33.7 (9.9) | 32.4 (10.8) | 36.7 (6.6) | 0.012 |

Values in table are mean (SD), number (%), or median (interquartile range).

BMI, body mass index; GFR, glomerular filtration rate; LDL, low-density lipoprotein; HDL, high-density lipoprotein.
